# *Anopheles* larval ecology and physicochemical characterization of larval habitats in Dire Dawa: an area colonized by *Anopheles stephensi* in Eastern Ethiopia

**DOI:** 10.1186/s12936-026-06082-w

**Published:** 2026-08-01

**Authors:** Ephrem Abiy, Teshome Degefa, Meshesha Balkew, Hailu Merga, Deneke Zewdu, Alamayo Ayala, Harrysone Atieli, Ming-Chieh Lee, Guofa Zhou, Guiyun Yan, Delenasaw Yewhalaw

**Affiliations:** 1 School of Medical Laboratory Sciences, Institute of Health, Jimma University, Jimma, Ethiopia; 2 Tropical and Infectious Diseases Research Center (TIDRC), Jimma University, Jimma, Ethiopia; 3 Aklilu Lemma Institute of Health Research (ALIHR), Addis Ababa University, Addis Ababa, Ethiopia; 4 Department of Epidemiology, Institute of Health, Jimma University, Jimma, Ethiopia; 5 Department of Population Health and Disease Prevention, Joe C. Wen School of Population and Public Health, University of California at Irvine, Irvine, USA; 6 Tom Mboya University, Homa Bay, Kenya

**Keywords:** *Anopheles stephensi*, Larval habitat, Urban‒rural spectrum of landscape, Habitat physicochemical characteristics, *Anopheles* species diversity

## Abstract

**Background:**

Understanding mosquito larval ecology is crucial for effective vector control strategies. While much is known about *Anopheles* larval habitat distribution, the impact of landscape (urban vs. rural) on *Anopheles stephensi* larval distribution and population dynamics in Ethiopia remains unclear.

**Methods:**

A longitudinal study was conducted from February 2023 to December 2024 in aquatic habitats in urban, peri-urban, and rural areas of Dire Dawa City Administration, eastern Ethiopia. Mosquito larvae and pupae were collected and reared in a field insectary until adult emergence. The female *Anopheles* were morphologically identified and further confirmed via polymerase chain reaction (PCR). The physicochemical parameters of the larval habitats were measured via a HANNA multiparameter water probe. Statistical analyses included general linear models, ANOVA, logistic regression, paired sample t tests, chi-square tests, principal component analysis and correlation analyses to assess larval presence, density, and distribution in relation to water physicochemical parameters across the three ecological settings.

**Results:**

A total of 23,526 larvae and 1808 pupae of *Anopheles* mosquitoes were collected from 856 man-made habitats (bricks, plastic sheets, steel drums, tire tracks, canal ditches, and barrels) and 53 natural habitats (river edges, animal hoof prints, ponds, and swamps) in urban, peri-urban, and rural areas. Uncovered cemented cisterns were the main human-made larval habitats; river edges were the main natural habitats. *Anopheles* larvae were absent in the steel drums and plastic barrels at the rural sites. *Anopheles* larval density significantly differed across the different ecological settings (F = 7.8, df = 1, 908, p = 0.005) and among the different larval habitat types (F = 326.2, df = 1, 907, p < 0.001). Among the 2934 reared adults, 74.8% (2194/2934) were *An. stephensi*, 21.7% (636/2934) were *An. arabiensis*, and 3.0% were other species (*An. pharoensis*, *An. coustani*, *An. amharicus*, *An. pretoriensis*). *An. stephensi* shares habitats with *An. arabiensis* and *An. amharicus*. Larval presence was significantly associated with brick factory proximity (χ2 = 23.8, df = 5, p < 0.001), land use/surrounding environment (χ2 = 32.6, df = 13, p = 0.002), vegetation (χ2 = 27.1, df = 12, p = 0.008), shade coverage (χ2 = 25.9, df = 15, p = 0.039), substrate type (χ2 = 34.1, df = 6, p < 0.001), and competitor/predator presence (χ2 = 14.6, df = 1, p = 0.001). However, larval presence was not associated with the presence of larval interventions (χ2 = 1.3, df = 1, p = 0.250). Water pH (r = 0.26, n = 102, p < 0.050) and water pressure (r = 0.21, n = 102, p < 0.050) were the only water physicochemical parameters that were positively correlated with *An. stephensi* presence.

**Conclusion:**

*Anopheles stephensi* was dominant in urban areas but was also present in rural areas. Existing larval source management methods do not prevent *Anophel*es from occurring in many habitats, indicating an urgent need for improved strategies.

**Supplementary Information:**

The online version contains supplementary material available at https://doi.org/10.1186/s12936-026-06082-w.

## Introduction

*Anopheles* larval habitat characterization refers to the process of identifying and describing the environmental conditions where *Anopheles* mosquitoes lay their eggs and where their larvae develop [1]. These habitats are typically shallow bodies of water, such as ponds, swamps, streams, rice fields, and containers, which can vary in size and water chemistry. The characterization includes factors such as water depth, vegetation, temperature, pH, salinity, and the presence of organic matter, as these conditions influence the survival and development of larvae [2–4]. *Anopheles* larval habitats may differ among different ecological settings, e.g., urban, peri-urban and rural areas [5,6]. Understanding *Anopheles* larval ecology, such as larval distribution and population dynamics in different eco-settings, is crucial for targeted malaria control, especially for *Anopheles stephensi*, since it thrived in urban environments since it invaded East Africa [6,7].

Each malaria vector has its own larval habitat preference for oviposition, which might be associated with the genetic composition or environmental parameters that impose behavioral variation [8].

The main malaria vector *An. gambiae* complex prefers to breed in the clean water of temporary rain pools, hoof prints, natural pockets of rivers, and saltwater (some species) [9–12]. The *An.funestus* groups breed in emergent vegetation and year-round river sides, breed in rice fields, and under different irrigation schemes [12–14]. However, invasive *An. stephensi* breeds in artificial small containers, jars, bricks, plastic sheets, barrels, iron tankers, steel drums, discarded tires, and ditches, sometimes even in polluted water and fountains [7,15]. Although *An. stephensi* habitats have been extensively studied in Ethiopia [15–20], the differences in the distributions of habitat types and larval densities among different landscape settings have not been explicitly explored.

Additionally, the increase in aquatic habitat biotic and abiotic factors affects *Anopheles* female oviposition and larval development in both *An. stephensi* and other *Anopheles* species [1,21]. These factors include habitat physical characteristics such as vegetation coverage, turbidity and water temperature; water chemical properties such as pH value; dissolved oxygen and salinity; and the presence of predators and competitors [22–26]. However, no studies have demonstrated a correlation between water physicochemical parameters and *Anopheles stephensi* larval abundance in Ethiopia.

Finally, larval source management (LSM) has a proven track record in malaria control [27–29]. Historical successes in Brazil, Egypt, and parts of Asia have relied heavily on environmental management [30]. More recent studies have shown that larviciding via biological agents such as *Bacillus thuringiensis israelensis* (Bti) can reduce larval and adult vector densities by up to 90% in Kenya [27]. In Ethiopia, larval source management is assumed to be a supplementary strategy in addition to the principal vector control interventions using insecticide-treated nets and indoor residual spraying [31]. PMI-supported larviciding programs have been implemented in eight cities in Ethiopia for *An. stephensi* larval control. However, some laboratory and field evaluations have shown the high efficacy of Bti and Sumi Larv 2MR (biological) based larviciding in reducing *An. stephensi* larval density [32,33]. The efficacy of larvicide in eliminating *An. stephensi* larvae in treated habitats needs to be further assessed.

The aims of this study were to examine differences in *Anopheles* larval habitat distributions and larval population dynamics along the urban to rural spectrum and to assess the effects of the physico‒chemical properties of *An. stephensi* larval distributions in East Ethiopia. The results provide essential evidence for targeted *An. stephensi* larval control.

## Methods

### Study area

**Fig. 1:**
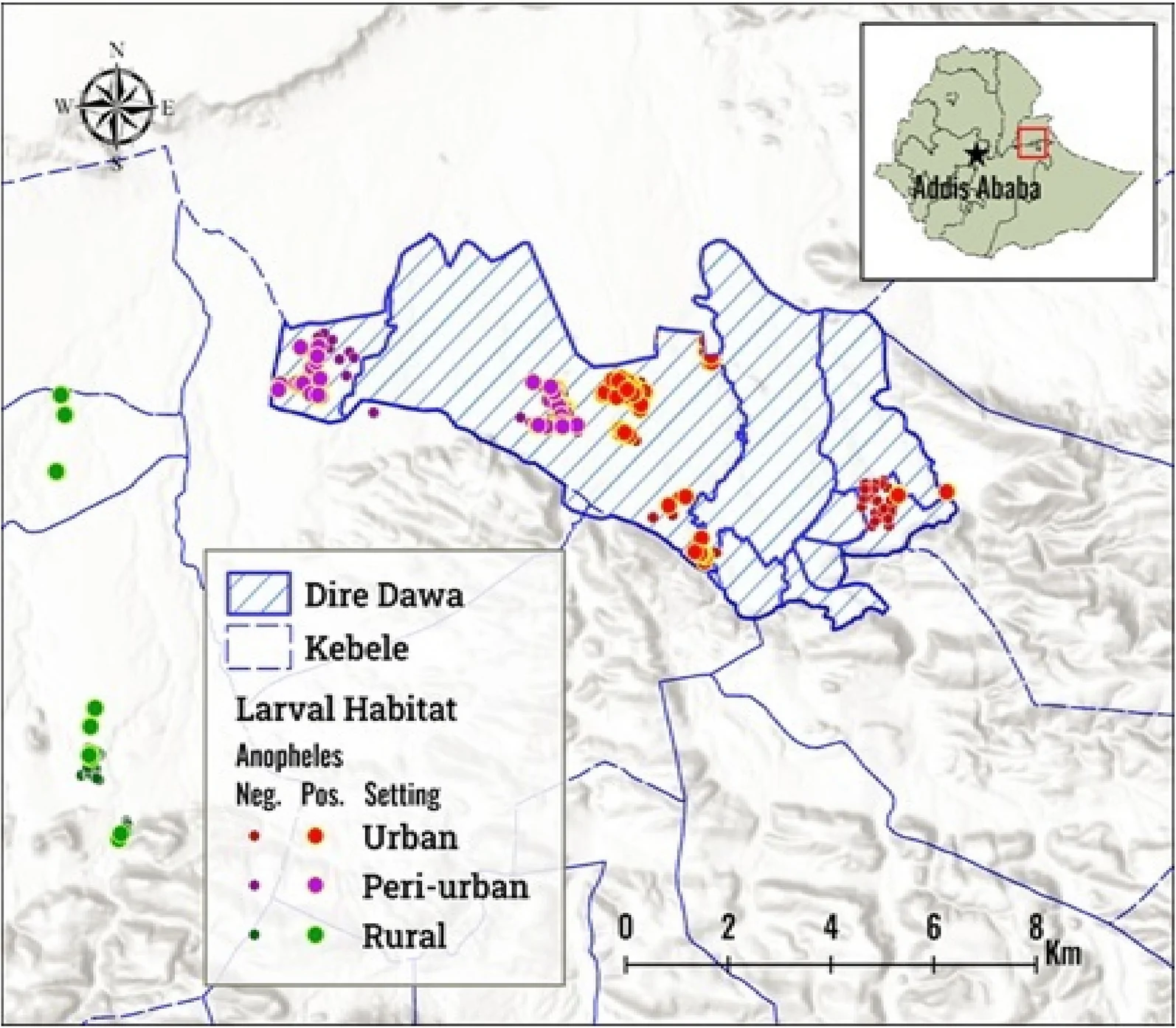
Map of the study area in Dire Dawa City Administration, 2024. This map was created with ArcGIS Pro 3.5 by Esri. (www.esri.com)

The study was conducted in three ecological settings of the Dire Dawa City Administration (515 km east of Addis Ababa), which included urban, per-urban and rural settings. The urban settings considered for this study were Dire Dawa University, the GTZ and Mermersa. The peri-urban sites were those around the industrial zone, including the villages of Melka-Jebdu and Jerba. The rural study sites were Aseliso, Hulahulul and Boren. The rural sites were agricultural sites with grass land and little vegetation coverage. (Fig. 1) [34]. During the study, larval source management was implemented jointly by the President’s Malaria Initiative (PMI) and the Ministry of Health of Ethiopia at urban and peri-urban sites [35].

The study area has seasonal malaria transmission, with *Plasmodium falciparum* and *P. vivax as* the main parasite species, and the vectors include *Anopheles arabiensis* and *An. phareonsis*. Recent studies reported the occurrence of *An. stephensi* as an additional vector [36,37].

### Immature mosquito collection and larval habitat characterization

Aquatic larval habitats were surveyed monthly from February 2023 to December 2024. Data were collected from house surveys, and prior to data collection, verbal and informed consent were obtained from the owners of the house. Ethical clearance was obtained from the Jimma University institutional review board (JUIH/IRB/236/25), and a support letter was also obtained from the Dire Dawa City Administration Health Bureau. Larval surveys were conducted following the standard WHO protocol, and mosquito samples were collected via a larval dipper with a capacity of 350 ml [38,39]. Twenty dips were taken from each habitat by dipping on different sides of the habitat for large habitats. For small habitats, collections were made with as many dippers as possible. Each larval instar and pupa were counted and recorded. The environmental and habitat information collected included geographic coordinates of the habitat, ecological setting (urban, peri-urban and rural), habitat type (Artificial and Natural) and picture of the habitat on the spot, source of water (tap, rain, or river), distance from the household, land use (surrounding environment of the habitat), shade status/degree of exposure to sunlight, water flow, substrate type, distance from brick making, distance from the nearest resting habitat and frequency of utilization, presence of canopy cover, presence of intervention, type of intervention and presence of predators. Mosquito immatures were classified as *Anopheles*, *Aedes* or *Culex* on the basis of morphological observations. The species composition of *Anopheles* mosquitoes was determined from adults reared from larvae or pupae. All the data collected were entered into an open data kit (ODK).

### Molecular species identification

The collected adult *Anopheles* were identified first to species via the morphological keys of Coetzee et al. 2020 and Glich et al. 1995 [40,41]. Those identified as *An. gamabiae* s.l. were further re-identified via species-specific PCR. Real-time PCR was also employed to confirm that *An. stephensi* at the Genomics Laboratory of Jimma University Tropical Infectious and Diseases Research Center (TIDRC).

### DNA extraction and amplification

DNA extraction was performed via both the Chelex and Extracta protocols [42]. PCR amplification was carried out according to the methods of Scott et al. [43] via the use of species-specific primers for *An. arabiensis* (AR: 5′-AAG TGT CCT TCT CCA TCC TA-3′) and *An. amharicus*, formerly *Anopheles quadriannulatus* B (QD: 5′-CAG ACC AAG AGA GAT GGT TAG TAT-3′) [11]. *Anopheles gambiae* (GA: 5′-CTG GTT TGG TCGGCA CGT TT-3′) and a universal primer (UN: 5′-GTGTGC CCC TTC CTC GATGT-3′). The amplicon was subsequently loaded on a 2% agarose gel stained with ethidium bromide and subjected to gel electrophoresis. *An. arabiensis* from the Sekoru insectary colony and *An*. *amharicus* from the Arjo area were used as positive controls [44].

### Physicochemical analysis of larval habitats

Physicochemical parameters of each habitat were measured via a HANNA (H198184) instrument with multiple parameters (Multiparameter Meter, Hanna Instruments Inc., Woonsocket, Rhode Island, 02895, USA) after proper calibration of the instrument with the manufacturer’s probe solutions, which were provided together with multiple parameters. Measurements, including acidity and alkalinity (pH/mV), oxidative reduction potential (ORP), electrical conductivity (EC), total dissolved solids (TDS), resistivity, salinity, seawater, dissolved oxygen (DO), and atmospheric pressure and temperature, were taken from 102 larval habitats by dipping the probe in the water (in situ) while on the ground for 100 sites, but for the remaining two animal hoof-print habitats where the amount of water on the surface was high, the water was collected in plastic tubes (100 ml) where each sample tube had an identification code and number, and readings were taken once per habitat. To avoid cross-contamination, the probes were rinsed in distilled water between sites.

### Statistical analysis

The data were entered into SPSS version 27, and descriptive analysis was used to compare the frequency, distribution, and magnitude of *Anopheles* larvae at the urban, peri-urban and rural interfaces. Multiple regression analysis was used to compare the effects of the physicochemical variables of the habitats on *Anopheles* larva presence/absence and larval density. Principal component analysis (PCA) was performed, and Spearman’s correlation coefficient was used to determine the correlations among water physicochemical parameters and larval presence and density. Cross tabs and Pearson’s chi-square tests were used to compare the associations of the presence/absence of predators and the presence/absence of interventions with larval presence and density. Generalized linear models and ANOVA were used to indicate the associations between mean larval density and habitat type in each ecological setting. Larval density was calculated as the number of larvae/pupae per 20 dips per habitat.

## Results

### Larval habitat abundance and indices

**Fig. 2:**
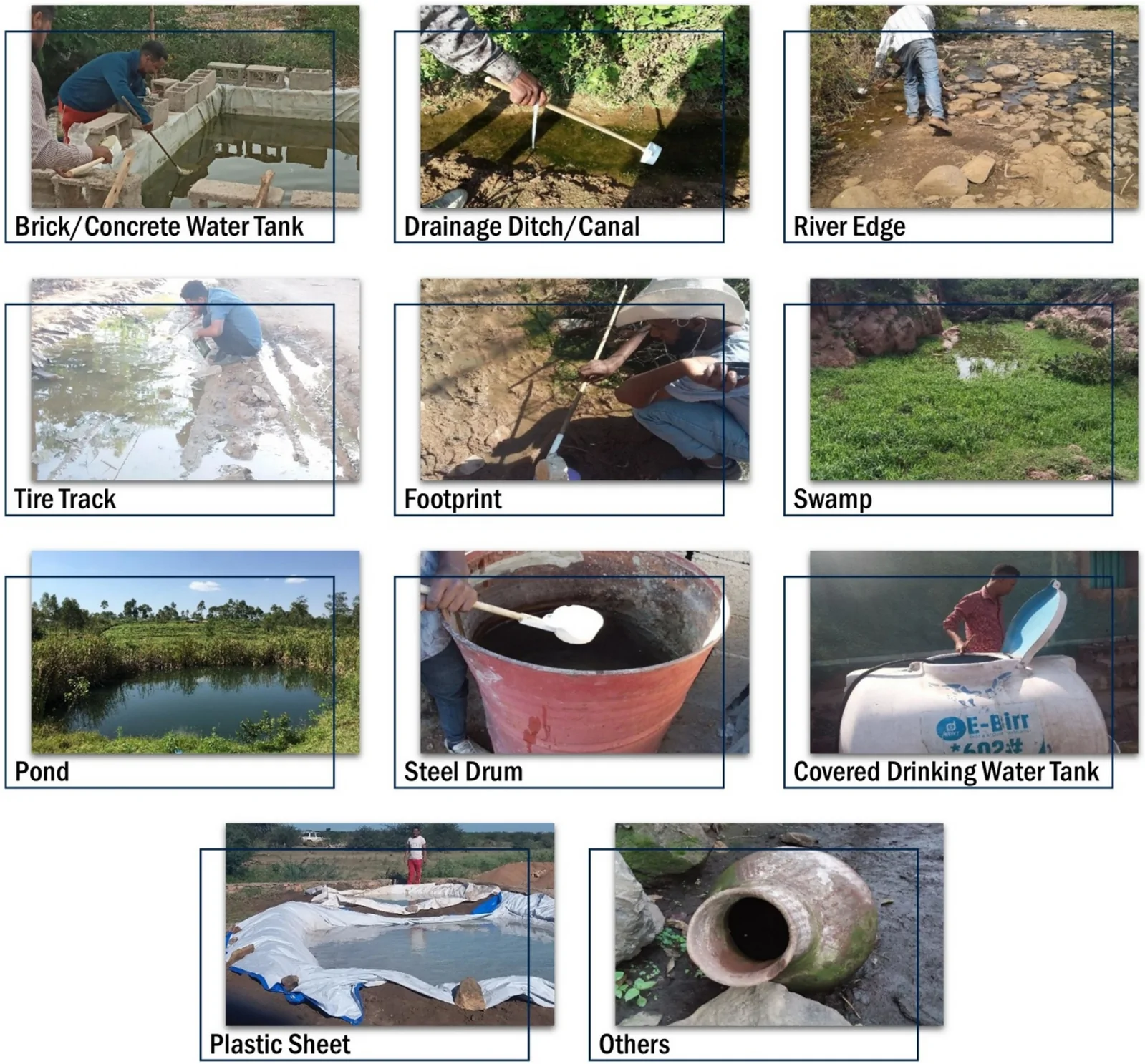
Types of larval habitats in Dire Dawa City Administration

A total of 909 habitats were surveyed in Dire Dawa, of which 856 (94.2%) were artificial breeding habitats, such as brick/concrete water tankers, plastic sheets, plastic water tankers (barrels), steel drums, canal ditches and tire tracks, whereas the other habitats were natural habitats, such as river edges, animal hoof prints, ponds and swamps (Table 1) (Fig. 2).

**Table 1:** Larval habitat abundance, proportion and larval presence positivity in urban, peri-urban and rural sites in Dire Dawa City Administration (2024)

Habitat type	n (%)	Positive for *Anopheles* (%)
Brick concrete water tanker	447 (49.2)	176 (39.4)
Barrel	82 (9.0)	13 (15.9)
River edge	26 (2.9)	15 (57.7)
Tire tracks	6 (0.7)	4 (66.7)
Foot print	11(1.2)	9 (81.8)
Swamp/Pond	16 (1.8)	8 (50.0)
Steel drum	25 (2.8)	5 (20.0)
Covered drinking water tanker/Birca	60 (6.6)	8 (13.3)
Plastic sheet	221(24.3)	58 (26.2)
Other (small water containers, pipe, foundation, septic tank)	15 (1.7)	2 (13.3)
Total	909 (100)	298 (32.8)

In Dire Dawa, brick cisterns were the predominant habitat type at 447/909 (49.2%), followed by plastic sheets at 221/909 (24.3%). *Anopheles* larvae were found in 298 (32.8%) aquatic habitats in Dire Dawa (Tables 1 and 2).

**Table 2:** Aquatic habitats in urban, peri-urban and rural ecologies in Dire Dawa

Setting	# Aquatic habitats surveyed (%)	# *Anopheles* positive habitat (%)	*Anopheles stephensi* positive habitats (%)	Other *Anopheles* positive habitats (%)
Urban	382 (42.0)	133 (34.8)	131 (98.5)	2 (1.5)
Peri-urban	307 (33.8)	108 (35.2)	105 (97.2)	3 (2.8)
Rural	220 (24.2)	57 (25.9)	1 (1.8)	56 (98.2)
Total	909 (100)	298 (32.8)	237 (79.5)	61 (20.5)

Nine hundred nine aquatic habitats were found, with 382 (42.0%) in urban sites, 307 (33.8%) in peri-urban sites and 220 (24.2%) in rural sites.

A total of 23,526 larvae and 1808 pupae of *Anopheles* mosquitoes were collected from 909 habitats in urban, peri-urban, and rural areas of Dire Dawa.

**Fig. 3:**
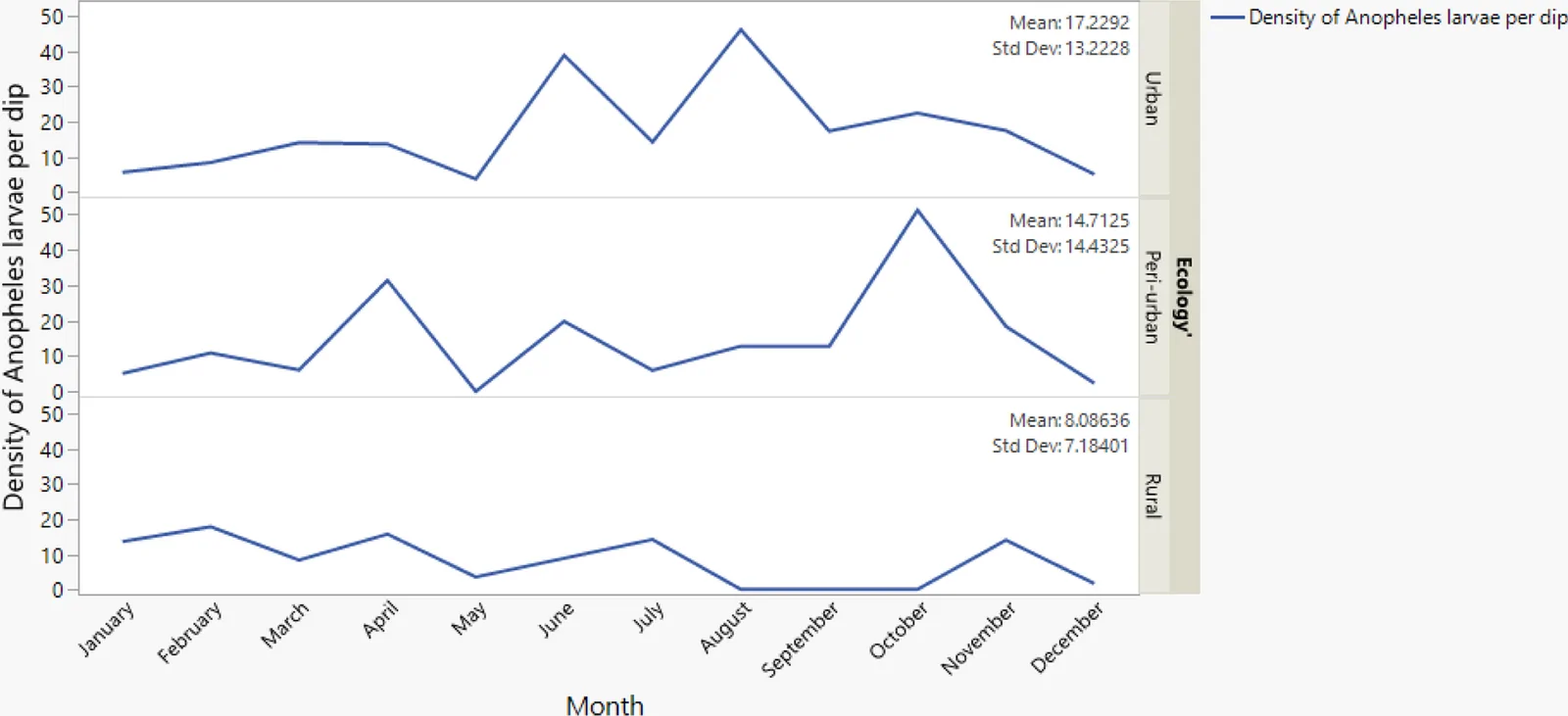
Mean *Anopheles* larval density by month and ecological setting in Dire Dawa, Ethiopia (2024)

The monthly mean *Anopheles* larval density was the highest in urban areas (mean ± standard deviation (SD) of 17.2 ± 13.0 larvae/dip), followed by that in peri-urban areas (14.7 ± 14.4 larvae/dip) and that in rural areas (8 ± 7.1 larvae/dip) (Fig. 3). Generalized linear models indicated that *Anopheles* larval densities were significantly different in the different ecological settings (F = 7.8, df = 1, 908, p = 0.005) and in different types of larval habitats (F = 326.2, df = 1, 907, p < 0.001).

Paired-samples t tests revealed a significant difference in the density of *Anopheles* larvae across months (t = − 3.3, df = 34, p = 0.003), with a mean difference of − 6.98 larvae per dip (95% CI − 11.3–2.6) and a medium effect size (Cohen’s dₚ = 0.6), indicating a moderate seasonal fluctuation in larval abundance. Similarly, the paired-samples t test revealed that larval density differed significantly between ecological categories (t = − 5.4, df = 34, p < 0.001), with a mean difference of − 11.52 larvae per dip (95% CI − 15.9 to  − 7.2) and a large effect size (Cohen’s dₚ = 0.9). A stronger effect was observed across ecological settings, which suggests that ecological characteristics exert a greater influence on *Anopheles* larval density than does seasonal variation alone.

In urban sites, where larval density was estimated to be constant throughout the year, the monthly *Anopheles* larval density was high in August and lowest in January. At the peri-urban sites, the *Anopheles* larval density was highest in October and absent in May. At the rural sites, larval density peaked in February and was absent from June to October (Fig. 3), indicating that there was a significant association between larval density and habitat stability across months (*t* = 7.4, n = 34, *p* < 0.001). In some months, the larval habitats dried, and no larvae were found, suggesting that temporal or ecological factors together influence larval dynamics.

**Fig. 4:**
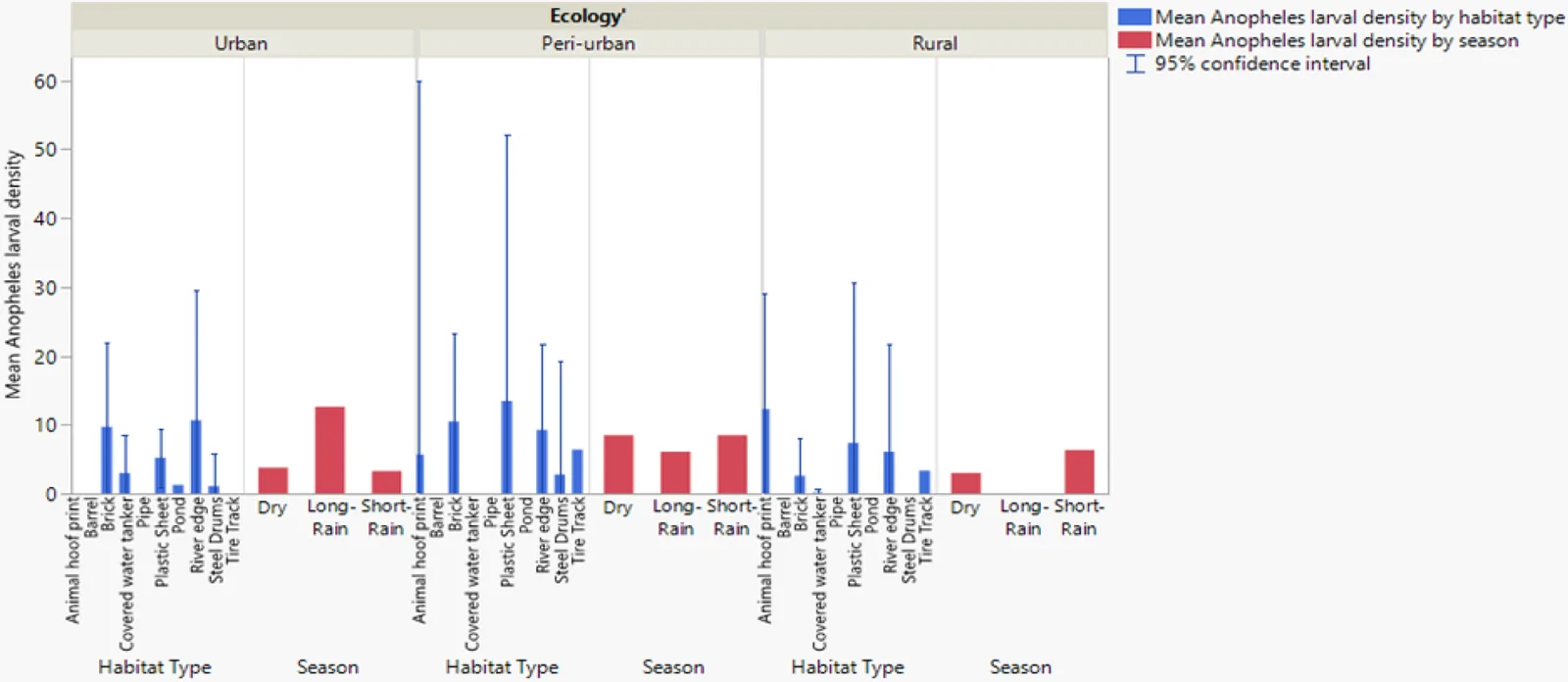
Mean *Anopheles* larval density by season, habitat type and ecological setting in Dire Dawa City Administration, Ethiopia (2024)

The mean larval density was highest at the peri-urban site in the artificial brick (51 larvae per dip), followed by natural (river edge) habitat type at the urban site (46 larvae per dip), and lowest in the plastic sheet habitats at rural and urban sites (0 per dip/habitat) (Fig. 4).

There was a significant difference between ecological settings (urban, peri-urban, and rural) and habitat types (ANOVA, F = 529.5, df = 1, 907, p < 0.001). Seasonal variations, specifically dry and long rainy seasons, strongly affect larval abundance and density (t = − 4.9, df = 77, p < 0.001).

As the construction of houses and production of bricks are common in the urban and peri-urban areas of Dire Dawa, the *Anopheles* larvae population density was high throughout the year in artificial water storage areas, regardless of the seasonal variation in the urban and peri-urban ecologies (Fig. 4). However, in rural areas, *Anopheles* larval density increased following the short rainy season (Fig. 4).

### *Anopheles* risk factors

Approximately 80.5% (732/909) of the aquatic habitats were located less than 50 m from nearby living houses, and 65.0% (589/909) of the habitats were 0–50 m away from brick-making houses. The results of logistic regression analysis indicated that man-made/artificial habitats and habitats surrounded by grassland were significant predictors of the presence of *Anopheles* larvae/pupae (Table 3).

**Table 3:** Logistic regression analysis for the assessment of predictors of mosquito larval occurrence in urban, peri-urban and rural areas of Dire Dawa City Administration, Ethiopia

Variable	Estimate	x^2^	p value	Lower 95%	Upper 95%
Eco setting [peri-urban]	0.249	1.34	0.2469	− 0.172	0.675
Eco setting [rural]	− 0.251	1.03	0.3103	− 0.747	0.228
Habitat category [man made]	2.222	5.76	0.0164	0.823	4.805
Landuse2 [construction]	0.199	0.19	0.6608	− 0.638	1.148
Landuse2 [farmland]	0.407	0.7	0.4023	− 0.534	1.386
Landuse2 [grassland]	1.504	4.45	0.0349	0.165	3.019
Landuse2 [house]	− 0.588	1.05	0.3058	− 1.717	0.557
Landuse2 [road]	− 1.943	1.28	0.2587	− 5.645	1.139
Vegetation cover	− 0.008	0.23	0.6321	− 0.041	0.027
Shade coverage	0.001	0.01	0.942	− 0.018	0.018
Larval source management (LSM)	− 0.525	0.88	0.3494	− 1.693	0.540
Distance to house	− 0.004	2.26	0.1325	− 0.010	0.001
Distance to resting	0.000	0	0.988	− 0.005	0.005
Distance to brick making	− 0.003	1.69	0.1932	− 0.009	0.001
Habitat length	− 0.031	0.86	0.3531	− 0.107	0.032

**Fig. 5:**
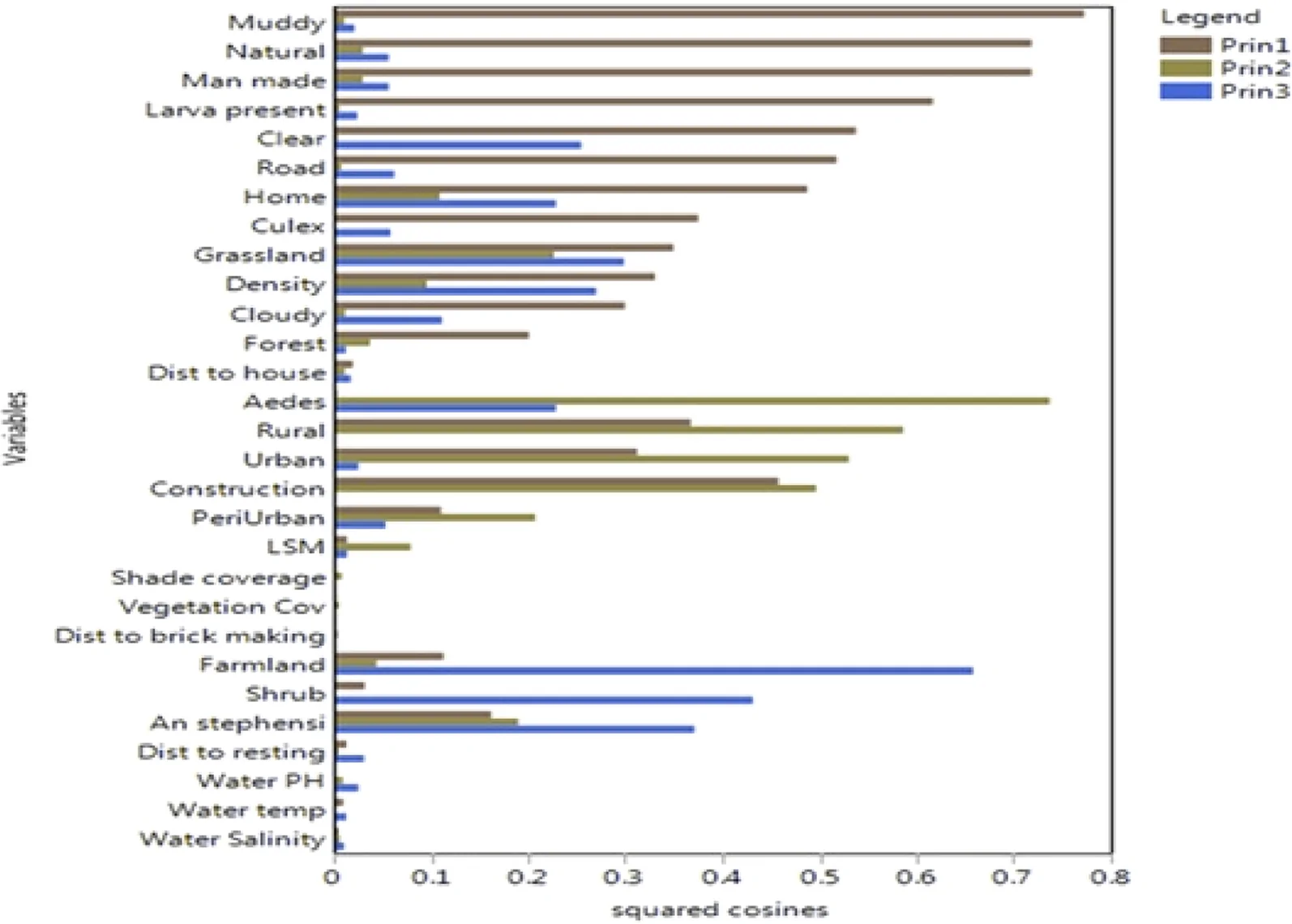
Variables grouped via PCA. Variables grouped together are correlated. PCA 1 (dark red bars) includes *Anopheles* and *Culex* larval presence, PCA 2 (dark yellow bars) includes *Aedes* larval density, and PCA 3 (blue bars) includes*Anstephens*. *Anstephens* presence

Both natural and man-made habitats presented the same correlation with *Anopheles* and *Culex* larval presence and *Aedes* larval density. However, rural sites were more highly correlated with *Aedes* larval density than were urban and peri-urban sites. Conversely, the presence of *An. stephensi* larvae was more strongly correlated with urban and peri-urban sites than with rural sites. Man-made habits are common at construction sites, brick factories, and urban expansion sites, and every household owns at least single water storage sites that support the breeding of *Anopheles*. Similarly, the results of the PCA indicated that positive correlations between land use (surrounding environment) and larval presence, habitat within the Grass-land environment, farmland, and the availability of shrubs and within or near households were correlated with the presence of *Anopheles*, *Culex and Aedes* larvae (Figs. 5 and 7).

The results of the principal component analysis (PCA) of the water samples (n = 102) used for physicochemical analysis indicated that the presence of larvae/pupae was positively correlated with pH (r = 0.26, n = 102, p < 0.050) and water pressure (r = 0.21, n = 102, p < 0.050). Other water physicochemical parameters were not associated with larval presence/abundance or density.

**Fig. 6:**
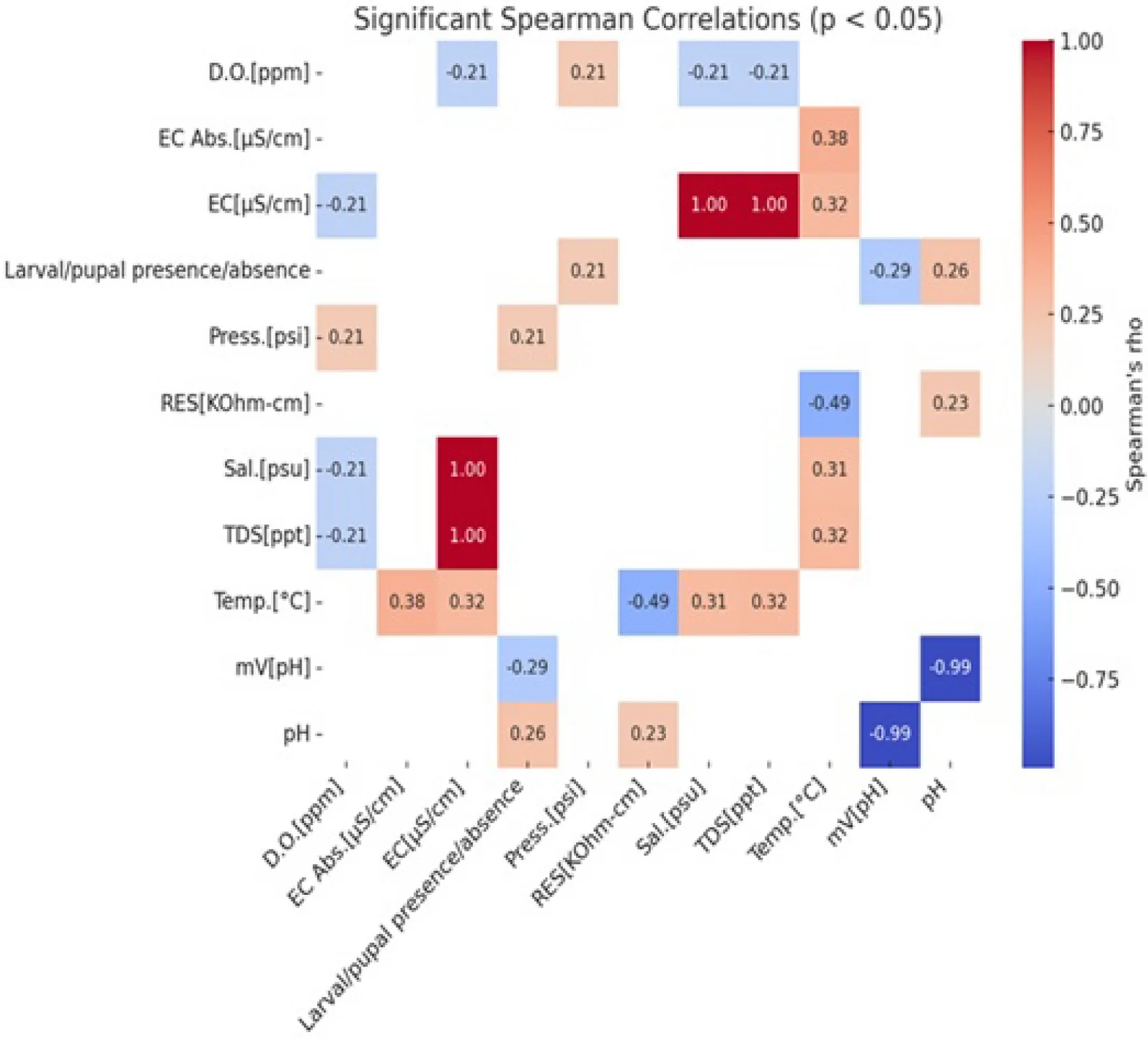
Results of Spearman’s correlation among predictors and the occurrence of mosquito immatures

The PCA also revealed that there was a significant positive correlation between temperature and conductivity (EC) (r = 0.31, p = 0.020), total dissolved solids (TDS) (r = 0.38, p = 0.020) and salinity (r = 0.38, p = 0.020) (Fig. 6).

In addition, EC, TDS and salinity were positively correlated (r = 1, p = 0.00). Additionally, dissolved oxygen (DO) and water pressure (psi) were positively correlated (r = 0.21, p = 0.040). However, temperature and resistivity had a negative correlation (r =− 0.49, p = 0.00), and pH and mvpH also had a negative correlation (r = − 0.99, p = 0.00). In addition, there was no significant correlation between temperature and dissolved oxygen or between dissolved oxygen and salinity (p > 0.05). The oxidative reduction potential (ORP) was the variable that had no significant correlation with all the independent variable (Fig. 6).

**Fig. 7:**
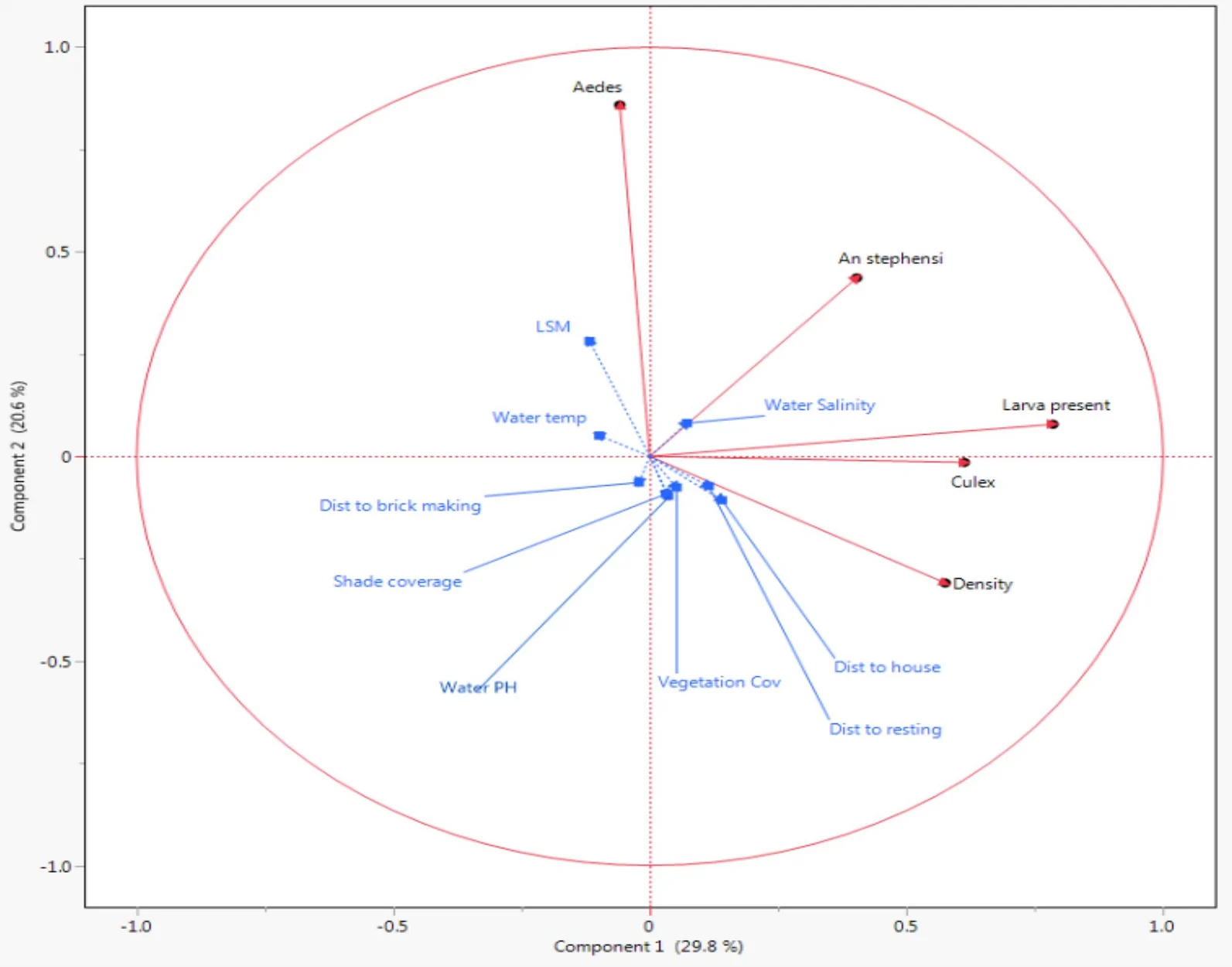
Correlation of the environmental and physicochemical parameters with the presence and density of mosquito immatures

The PCA results also revealed that *Anopheles* larval/pupal density was positively correlated with shorter distances to living houses, distances to resting places, availability of brick-making, vegetation cover, shading, pH and salinity (Fig. 7).

The Pearson’s chi-square test revealed no significant association between the presence of predators or competitors and larval presence (χ^2^ = 14.6, df = 1, *p* < 0.0001). Predator occurrence was similarly low whether larvae were present or absent.

Interestingly, the Pearson’s chi-square test indicated that there was no significant association between the presence of the intervention (larviding) and larval presence (χ^2^ = 1.3 df = 1, *p* = 0.261), and the presence of the intervention did not affect larval presence (*p* > 0.05).

As depicted in Figs. 5 and 7, water salinity was positively correlated with the presence of *Anopheles stephensi*.

The analysis of water salinity revealed that the majority of samples (88.3%) fell within the valid or normal salinity range, indicating predominantly fresh water conditions across the study area. Only 11.7% of the 873 samples presented measurable salinities above 0 ppt. Among these, slightly saline water (2–3 ppt) was the most common, accounting for 5.6% of the samples, followed by low salinity levels between 0–1 ppt (2.4%). Higher salinity categories were much less common, with only 1.8% of samples in the 4–5 ppt range and less than 1% exceeding 6 ppt. Extremely saline samples (> 30 ppt) represented just 0.3% of the total samples (Table 4).

**Table 4:** Results of water salinity measurements of *Anopheles* larval habitats in Dire Dawa City Administration, Ethiopia

Salinity (ppt)	N (%)	Category
0–1	21(2.4)	Very low salinity (freshwater)
2–3	49 (5.6)	Slightly saline (brackish tendency)
4–5	16 (18)	Moderately saline
6–7	6 (0.7)	Noticeably saline
8–9	2 (0.2)	Highly saline
10–14	5 (0.6)	Very saline—approaching seawater levels
> 30	3 (0.3)	Extremely saline (seawater or hyper-saline)

Moreover, water temperature and water pH were among the strong predictors of the presence of *An. stephensi* larvae (Fig. 7). The water physicochemical analysis indicated that the water temperature of the larval habitat ranged from 19 °C to 34 °C. The water temperature for 80.4% (82/102) of the larval habitats ranged from 20 to 27 °C, 16.7% (17/102) of the larval habitats had water temperatures ranging from 28 to 34 °C, and for the remaining 2.9% (3/102) of the habitats, the water temperature recorded was 19 °C.

The water pH values were measured and categorized into three categories: acidic (pH value < 7), neutral (pH value = 7) and alkaline (pH > 7). The results indicated that 81.4% (83/102) of the habitats were alkaline, 15.9% (16/102) were neutral and 2.9% (3/102) were acidic (Table 5).

**Table 5:** Results of water pH measurements of larval habitats in Dire Dawa City Administration, Ethiopia

pH	N (%)	Std. Error	95% Confidence interval	pH
			Lower	Upper
Acidic	3 (2.9)	1.7	0.0	6.9
Neutral	16 (15.7)	3.7	8.8	22..5
Alkaline	83 (81.4)	3.9	73.5	89.2
Total	102 (100)	0.0	100.0	100.0

Five types of substrates were considered (gravel, sand, mud, concrete and plastic cloth), and the results of the study revealed that 50.9% (52/102) of the habitat substrates were made of concrete and that 25.5% (26/102) of the habitat substrates were made of plastic cloths/containers. The chi-square test indicated that there was a statistical association between larval presence and substrate type (χ^2^ = 34.1, df = 6, *p* < 0.01).

### Species diversity and evenness

A total of 2934 adult *Anopheles* mosquitoes emerged from immature mosquitoes at all sites, and *An. stephensi* was the predominant species at 74.8% (2194/2934), followed by *An. arabiensis* at 21.7% (636/2934), *An. pharoensis (*1.7%; 50/2934), *An. coustani* (1.3%; 38/2934), *An. amharicus* (0.3%; 9/2934) and *An. pretoriensis* 0.2% (7/2934), respectively (Supplement Table 1).

Further paired samples *t* tests revealed significant differences in species composition between habitat categories (man-made and natural). The abundance differed significantly between habitats (*t* = − 2.2, df = 13, *p* = 0.044), with man-made habitats having higher *Anopheles* abundance. Significant habitat-related differences were also observed in Shannon diversity (t = 10.0, df = 13, *p* < 0.001), species evenness (5.7, df = 13, *p* < 0.001), and species richness (t = 5.7, df = 13, *p* < 0.001), indicating greater diversity, more even species distributions, and higher species richness in the natural habitat category.

*Anopheles stephensi*, *An. arabiensis* and *An. amharicus* were found to share the same artificial habitats, more specifically, plastic sheets (in urban, peri-urban and rural areas), and in natural habitats, more specifically, a river, the Butuji River, found in Dire Dawa city.

Overall, natural habitats presented greater species diversity and richness than artificial habitats did (Supplement Table 1). In addition, habitats in rural areas presented greater species diversity and richness than peri-urban and urban areas did (Supplement Table 1).

### Molecular identification of *An. gambiae *s.l. and *An. stephensi*

Of the 645 *An. gambiae* s.l. adult (emerged from larval collections) samples examined via PCR, 98.6% (n = 636) were *An. arabiensis*, and 1.4% (n = 9) were *An. amharicus*. Real-time PCR (qPCR) was performed on 200 morphologically identified *An. stephensi* specimens, confirming a 100% (200/200) correct morphological identification rate. Different *Anopheles* species were found in different types of larval habitats but also co-exist in same habitat type and ecological settings. *An. arabiensis* and *An.stepehensi* co-existed in river edges in Urban and *An.amharicus* and *An.stephensi* were co-existed in plastic sheet habita type in rural study area (Supplement Table 2).

## Discussion

Our study indicated that *Anopheles* immature stages are abundant throughout the year regardless of the seasonal variation in rain, with peak larval densities in the dry season in all three ecological settings, which concurs with previous studies in Jijiga and Somali regions in East Ethiopia, where the climatic and ecological settings are similar to those of Dire Dawa [17]. This might be because both Dire Dawa city and Jigjiga town used similar man-made water containers at construction sites, brick factories and water reserves for household purposes. This expansion of urbanization and construction activities supported the breeding of immature stages in the dry season.

Moreover, *Anopheles* larval habitats were found less than 50 m from living houses, and brick-making houses were located 0–50 m from living houses. This finding has important implications for supporting the life cycle of *Anopheles* and maximizing the probability of human vector contact. This finding could help to implement targeted larviciding and larval source management in habitats that harbor immature stages throughout the year.

The other finding of our study was the coexistence of *An. stephensi* with *An. gambiae s.l.,* which shares both artificial and natural habitats and different ecological settings. *An. stephensi* is known to be a vector of malaria, mainly in urban settings and artificial habitats [7,24]; however, in our study, *An. stephensi* was found in rural areas in Dire Dawa and at river edges of the Legehare and Butuji rivers, the two known and permanent habitats in the city. These findings further highlight the expansion of *An. stephensi* to rural sites and its adaptation to natural habitats for breeding. Our findings coincide with those of a study performed in southeastern Iran, where *An. stephensi* was found at river edges with sandy substrates [25].

An unexpected result of our research in the study area was the presence of *An. amharicus* in Eastern Ethiopia, which has been reported in other parts of the country [11,45]. We found *An. amharicus* in the urban, peri-urban and rural ecologies of Dire Dawa for the first time in Eastern Ethiopia. Additionally, *An. coustani*, *An. pretoriensis and An. phareonis* were found in the study area*.*

These findings suggest that further investigations on the role of *An. amharicus* and other vectors in malaria transmission in Dire Dawa and their general bionomics, including their susceptibility to current insecticides in use, are important.

Water physicochemical analysis indicated that larval distribution and abundance were associated with water physicochemical parameters. In our study, pH and water pressure were the main predictors that were positively correlated with larval presence. Our study aligns with a study conducted in northern Iran, which revealed significant differences in physicochemical characteristics and larval density [46].

In contrast, our analysis of water salinity revealed that the majority of samples (88.3%) fell within the valid or normal salinity range and that 86% fell within the alkaline pH range, indicating predominantly fresh water conditions across the study area. Studies on the correlation of *Anopheles* larval density with physicochemical characteristics in Benin and southeastern Iran indicated that there was a high positive correlation of larval density with temperature [26], dissolved oxygen and salinity [25,26]. This difference might be because of the difference in the sources of water in the urban areas of Benin and southeastern Iran from our study area, where almost 98% of the sources of water for larval habitats were groundwater and where the chemical characteristics were within similar ranges.

In agreement with a study conducted by Mereta et al. [22], our study revealed a widespread distribution of *Anopheles* larvae in small man-made aquatic habitats; however, in contrast to the findings of Mereta et al. (2013) but smillar to Iran, our study revealed that *Anopheles* larvae were abundant in both natural and artificial habitats.

The year-round presence of larvae in artificial habitats indicated that larval density, distribution and abundance were not affected by seasonal rainfall, as long as the water supply continued for the purpose of construction, brick making and drinking. The expansion of *An. stephensi* to rural sites could be due to rapid urbanization, industrial expansion and rural construction/agricultural activities, which are accompanied by the construction of water storage in rural areas. The *An. stephensi* samples collected at rural sites in Aseliso (40 km away from Dire Dawa city) in a plastic sheet water reservoir habitat were good examples for explaining why An. stephensi was found there, i.e., the presence of *An. stephensi* in rural areas was strongly associated with man-made permanent habitat. However, the density and abundance of *An. stephensi* were higher at urban and peri-urban sites than at rural sites, which could be due to the abundant construction and brick manufacturing in urban and peri-urban settings.

### Limitations of the study

Physicochemical tests were performed in a cross-sectional way during one seasonal survey, and seasonal variation may affect water physicochemical properties, which in turn may affect the associations between water physicochemical properties and *Anopheles* larval presence and abundance. Larval rearing is one way to confirm the presence of specific mosquito species in a given aquatic habitat. However, this is not an ideal approach because adult mosquitoes do not emerge in many experiments; this not only affects the confirmation of the presence of specific species but also affects larval density estimates because we do not know the true emergence rate for different mosquito species. Another limitation is that the study was conducted in one city in the dry area of east Ethiopia where natural aquatic habitats are sparse, even in rural areas. Places such as Badu and Hawassa in the rift valley of Ethiopia, where there is either plenty of rainfall (e.g., Hawassa) or many natural habitats, such as lake shores and irrigation (e.g., Badu) and *An. stephensi* has been reported from both places, may provide different ecological settings, especially with more natural habitat, and may result in different associations between landscape settings, habitat water physicochemical properties, and *An. stephensi* ecology, e.g., presence and larval abundance.

## Conclusion

*Anopheles stephensi* was the predominant species found in the study area, followed by *An. arabiensis, An. amharicu, An. pharoensis, An. coustani and An. pretoriensis*. Cistern/bricks tankers were the most efficient habitat type at urban and peri-urban sites, followed by plastic sheet water reservoir habitats, whereas hoof prints and river basins under natural habitats were the most efficient habitat types at rural and urban sites, respectively. We report here the existence of *An. amahricus* for the first time in east Ethiopia, the expansion of *An. stephensi* in rural areas, breeding in natural habitats, and coexisting with *An. gambiae s.l.* The availability of brick-making and shorter distances from living houses in urban ecology were directly correlated with *An. stephensi* larval density. The habitat abundance and positivity of uncovered water tankers at urban and preurban sites could indicate the feasibility and proper implementation of larval source management.

## Supplementary Information

Supplementary Material 1

## Data Availability

The datasets used and/or analyzed during the current study are available from the corresponding author upon reasonable request.
