# Supplementary material for: Anopheles larval ecology and physicochemical characterization of larval habitats in Dire Dawa: an area colonized by Anopheles stephensi in Eastern Ethiopia
Source: Malar J. 2026 Aug 1;25:386. doi: 10.1186/s12936-026-06082-w (PMC13721734; doi:10.1186/s12936-026-06082-w)
Supplement: Supplementary file 1 [file 12936_2026_6082_MOESM1_ESM.docx]

Supplement Table 1 *Anopheles* mosquito species diversity, richness and evenness by habitat type in Dire Dawa City Administration, Ethiopia, 2024.

| Habitat | Species | Abundance | Proportion  (pi) | Lnpi | pi*Lnpi | Shannon  diversity index (H') | Species  eveness (J): H/LnS | Species  richness (S) |
| --- | --- | --- | --- | --- | --- | --- | --- | --- |
| Artificial | *An.stephensi* | 1873 | 0.91 | -0.1 | -1. | 0.1 | 0.3 | 3 |
|  | *An.arabiensis* | 185 | 0.09 | -2.4 | -0.2 | 0.22 |  |  |
|  | *An.amharicus* | 8 | 0.00 | -5.6 | -0.1 | 0.02 |  |  |
|  | *An.pretoriensis* | 0 | 0 | 0 | 0 | 0 |  |  |
|  | *An coustani* | 0 | 0 | 0 | 0 | 0 |  |  |
|  | *An.pharoensis* | 0 | 0 | 0 | 0 | 0 |  |  |
| *Total* |  | 2066 | 1 | 0 | 0 | 0.33 |  |  |
| Natural | *An.stephensi* | 321 | 0.37 | -1 | -0.4 | 0.4 | 0.6 | 6 |
|  | *An.arabiensis* | 451 | 0.52 | -1 | -0.3 | 0.3 |  |  |
|  | *An.amharicus* | 1 | 0.00 | -7 | -0.01 | 0.01 |  |  |
|  | *An.pretoriensis* | 7 | 0.01 | -5 | -0.04 | 0.04 |  |  |
|  | *An.coustani* | 38 | 0.04 | -3 | -0.14 | 0.14 |  |  |
|  | *An.pharoensis* | 50 | 0.06 | -3 | -0.16 | 0.16 |  |  |
| *Total* |  | 868 | 1 | 0 | 0 | 1.06 |  |  |

Supplement Table 2 Species, identification method and Larval habitat diversity in urban, peri-urban and rural settings, Dire Dawa City Administration

| Species | Ecology found | Habitat type | Identification mechanism |
| --- | --- | --- | --- |
| *An.stephensi* | All (urban, peri-urban and rural) | Man-made predominantly/River in town | qPCR and Morphological identification |
| *An.arabiensis* | All | Natural habitats/some artificial plastic sheet | PCR |
| *An.amharicus* | All | Artificial plastic sheet and cistern | PCR |
| *An.coustani* | Rural | Natural, river edge | morphology |
| *An.phareonsis* | Rural | Natural, pond | morphology |
